# A phase plane graph based model of the ovulatory cycle lacking the "positive feedback" phenomenon

**DOI:** 10.1186/1742-4682-9-35

**Published:** 2012-08-07

**Authors:** Sven Kurbel

**Affiliations:** 1 Dept. of Physiology, Osijek Medical Faculty, J Huttlera 4, Osijek, 31000, Croatia

## Abstract

When hormones during the ovulatory cycle are shown in phase plane graphs, reported FSH and estrogen values form a specific pattern that resembles the leaning “&" symbol, while LH and progesterone (Pg) values form a "boomerang" shape. Graphs in this paper were made using data reported by Stricker et al. [*Clin Chem Lab Med* 2006;44:883–887]. These patterns were used to construct a simplistic model of the ovulatory cycle without the conventional "positive feedback" phenomenon. The model is based on few well-established relations:

hypothalamic GnRH secretion is increased under estrogen exposure during two weeks that start before the ovulatory surge and lasts till lutheolysis.

the pituitary GnRH receptors are so prone to downregulation through ligand binding that this must be important for their function.

in several estrogen target tissue progesterone receptor (PgR) expression depends on previous estrogen binding to functional estrogen receptors (ER), while Pg binding to the expressed PgRs reduces both ER and PgR expression.

Some key features of the presented model are here listed:

High GnRH secretion induced by the recovered estrogen exposure starts in the late follicular phase and lasts till lutheolysis. The LH and FSH surges start due to combination of accumulated pituitary GnRH receptors and increased GnRH secretion. The surges quickly end due to partial downregulation of the pituitary GnRH receptors (64% reduction of the follicular phase pituitary GnRH receptors is needed to explain the reported LH drop after the surge). A strong increase in the lutheal Pg blood level, despite modest decline in LH levels, is explained as delayed expression of pituitary PgRs. Postponed pituitary PgRs expression enforces a negative feedback loop between Pg levels and LH secretions not before the mid lutheal phase.

Lutheolysis is explained as a consequence of Pg binding to hypothalamic and pituitary PgRs that reduces local ER expression. When hypothalamic sensitivity to estrogen is diminished due to lack of local ERs, hypothalamus switches back to the low GnRH secretion rate, leading to low secretion of gonadotropins and to lutheolysis. During low GnRH secretion rates, previously downregulated pituitary GnRH receptors recover to normal levels and thus allow the next cycle.

Possible implications of the presented model on several topics related to reproductive physiology are shortly discussed with some evolutionary aspects including the emergence of menopause.

## Introduction

**Figure 1:**
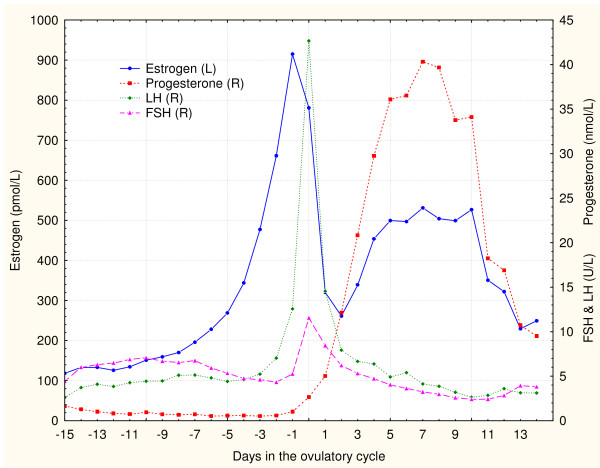
**Conventional time profile graphs of main ovulatory hormones reported by Stricker et al. [**[5]**], with separate left (L) and right (R) Y axes.**

**Figure 2:**
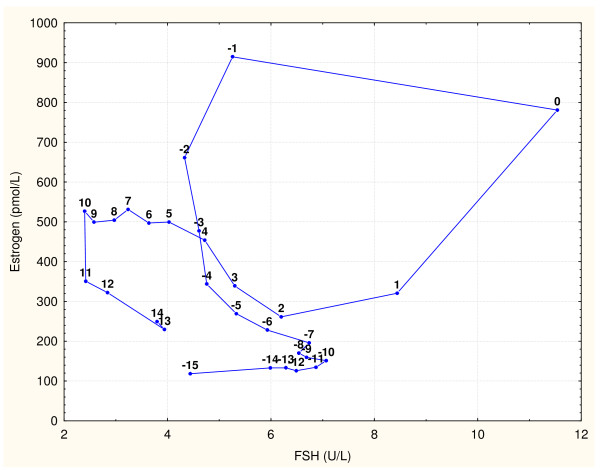
**FSH and estrogen values from Figure**1**, reported by Stricker et al. [**[5]**], shown as a phase plane graph.** Data points are labeled with days of the cycle. They form a pattern that resembles the leaning “*&*" symbol. Other published data produced very similar phase plane graphs [4,6-9], so it can be assumed that any set of ovulatory cycle data would provide similar patterns. This pattern is described in the The follicular phase to Lutheolysis.

**Figure 3:**
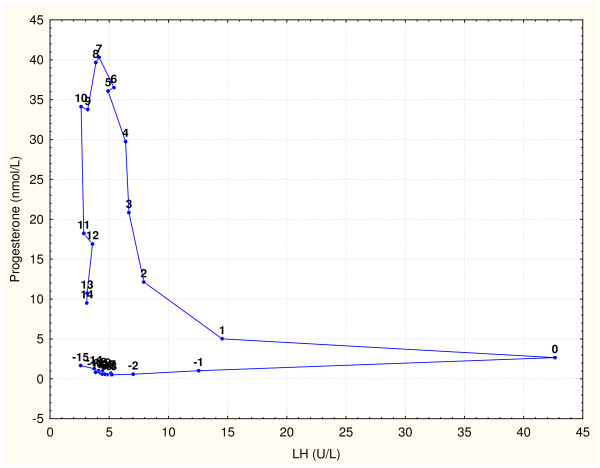
**LH and progesterone values from Figure**1**, reported by Stricker et al. [**[5]**], shown as a phase plane graph.** Data points are labeled with days of the cycle. They form a pattern that resembles a "boomerang" shape. Other cited data sets of hormonal values produced very similar phase plane graphs of these two hormones during the cycle This pattern is described in the The follicular phase to Lutheolysis.

Perfect timing of several hormone actions during the ovulatory cycle is essential for complex process of follicular growth, ovulation, and maintenance of *corpus luteum*. When presenting the complex dynamic of involved hormones, graphs often show time series data within an idealized 28 day ovulatory cycle, similar to Figure 1. This ideal cycle is usually centered on the ovulatory gonadotropin surge (on the day 0), so it starts with the −14 day and ends with the +13 day. It should be noted that the same data of time series can be presented as phase plane graphs (Figures 2 and 3) with data points labeled with days of the ovulatory cycle. This presentation type is potentially better suited to illustrate the intrinsic dynamic within the shown hormone pairs.

When explaining the sequence of events during the ovulatory cycle, most physiological textbooks are similar in description of two important phases of the ovulatory cycle: the ovulatory surge of gonadotropins (FSH & LH) and lutheolysis.

Often, more attention is payed to the mechanism of ovulation induction that happens after several days of raising estrogen blood levels in the late follicular phase. Then an almost paradoxical surge of gonadotropins, often described as the “positive feedback”, leads to the follicle rupture. In Boron & Boulpaep [1] this phenomenon is covered with several related statements “*Positive feedback of estrogens, progestins, and activins on the hypothalamic-pituitary axis is involved in the induction of this LH surge… the accelerated rate of increase in estradiol levels in the preovulatory phase sensitizes the gonadotrophs in the anterior pituitary to GnRH pulses*… *also modulate hypothalamic neuronal activity and induce a GnRH surge…the powerful positive feedback action of estradiol induces the midcycle surge of LH and, to a lesser extent, FSH.. Just before ovulation, the rise in estradiol secretion becomes more rapid and, by a positive feedback effect on the anterior pituitary, triggers a surge in LH, which causes ovulation. Also occurring just before ovulation is a smaller FSH surge that is triggered by a rise in progesterone as well as activin… Estradiol secretion by the dominant follicle increases rapidly near the end of the late follicular phase. This dramatic rise in circulating estradiol exerts positive feedback on the anterior pituitary and sensitizes it to GnRH. The net effect of a rising estradiol level is induction of the LH surge*…*.The LH surge appears to terminate in part as a result of rising levels of progesterone, via negative feedback, and in part as a result of loss of the positive feedback that is derived from estradiol. Depletion of gonadotropin stores in the anterior pituitary gland may also contribute to termination of the LH surge.*” (1).

Similar approaches can be found in other textbooks: “ …*LH secretion is held in check by the negative feedback effect of the rising plasma estrogen level. At 36 to 48 hours before ovulation, the estrogen feedback effect becomes positive, and this initiates the burst of LH secretion (LH surge) that produces ovulation…a moderate, constant level of circulating estrogen exerts a negative feedback effect on LH secretion, whereas during the cycle, an elevated estrogen level exerts a positive feedback effect and stimulates LH secretion*…” [2].

Often it is much less said about lutheolysis. In Boron & Boulpaep [1] it is covered with more elaboration: “*The high levels of progesterone, estrogens, and inhibins maximally suppress the hypothalamic-pituitary system. The result is that FSH and LH levels fall. As LH levels fall, the target of LH, the corpus luteum, rapidly diminishes its production of estrogen and progestins*… *the fall in estrogen and progestin levels diminishes the feedback inhibition on the hypothalamic-pituitary system, and gonadotropin secretion rises once again, thus beginning the next menstrual cycle.*”

Here presented model of the ovulatory cycle is an attempt to interpret both gonadotropin surges and lutheolysis without using the positive feedback phenomenon. The model is based on phase plane graphs of gonadotropins with estrogen and progesterone during a typical ovulatory cycle. This approach was first applied on digitalized values of FSH, LH, estrogen (E) and progesterone (Pg) during a typical 28-day ovulatory cycle, as reported by Chabbert Buffet et al. [3]. After that, the arithmetic means of FSH and estrogen values reported by Dighe et al. [4] were also tested with this approach. Median blood values of FSH, LH, estrogen and progesterone provided by Stricker et al. [5] were used for graphs in this paper. Figure 2 shows that data points in the phase graph of FSH with estrogen form a pattern that resembles the leaning “*&*" symbol, while Figure 3 shows that LH and progesterone form a "boomerang" shape phase plane pattern. Other published data [4,6-9] were used to produce very similar patterns, suggesting that any set of ovulatory cycle data results in similar hormonal interactions.

## Basic assumptions behind the proposed interpretation

Several assumptions were needed for the here proposed interpretation of complex phase plane patterns in Figures 2 and 3.

### GnRH rhythms and gonadal function

GnRH action is peculiar in two aspects [1,2]. First, it is normally secreted only in a regular rhythmic pattern and due to limited quantities and short half-life, secretion is inherently pulsatile. Second, the main GnRH target tissue, the anterior pituitary is exceptionally prone to downregulation of GnRH receptors (GnRHR). If the pituitary is overexposed to GnRH (or to a synthetic GnRH analogue), a transitory hypogonadism develops due to downregulation of pituitary GnRHRs that interrupts gonadotropin secretion. This unique susceptibility to downregulation must play some role in the physiologic gonadotropin secretion.

#### Inhibin, activin, leptin and other modulators of GnRH secretion

Several mediators are modulating the GnRH secretion. The pineal gland as a biorhythm regulator probably has some role in initiating puberty, but the body fat reserve seems more important in human reproduction. Information about the body fat reserve is probably mediated through leptin secretion from adipose tissue, particularly from the abdominal fat [10]. Only sufficient leptin exposure allows reproduction through hypothalamic leptin action. Low fat reserve in childhood and in anorectic patients prevent normal gonadotropin secretion.

The rhythm of GnRH secretion depends on hypothalamic action of sex hormone action, as shown in Table 1. In women regulation of GnRH secretion is complex. Two rhythms interchange during each of near 400 successive ovulatory cycles during the fertile period between puberty and menopause. Slower rhythm of GnRH secretion lasts during the follicular phase (from lutheolysis to preovulatory days), while the rhythm gets faster before ovulation and remains fast during the lutheal phase and slows again in lutheolysis [2]. Noting two things is important. The first is that shifts in GnRH secretion are expected only in women, they happen in lutheolysis and preovulatory and are compensated by changes in the availability of GnRH receptors within the following days. This might explain the unique susceptibility to downregulation of the GnRH pituitary receptors. The second important feature is that period of high GnRH secretion starts hours before the ovulatory surge and last till lutheolysis. This timing and duration prove that this shifting between two levels of GnRH secretion in female hypothalamus is surely important for the ovulatory surge, but it cannot explain it entirely. Based only on this shifting, one might expect only that in the late follicular phase gonadotropin secretion will rise and remain indefinitely stable on an elevated level, a situation quite analogous to the male setting in Table 1. No ovulation can be expected based only on increased GnRH secretion from the preovulatory days till lutheolysis.

**Table 1:** Here proposed different settings of leptin and GnRH actions during different phases of growth and aging and in anorexia nervosa

**Phases of growth & aging**			**GnRH rhythm and GnRHR availability**	
	**Leptin**	**GnRH**	**Female**	**Male**
Children	low	secretion	minimal secretion	
		receptors	normal or increased pituitary receptors	
Early pubertal	normal	secretion	intermediate secretion	
		receptors	normal pituitary receptors	
Healthy adult	normal	secretion	low secretion from lutheolysis through follicular phase till the preovuatory days	stable
			high secretion from preovulatory days till lutheolysis	
		receptors	slowly up-regulated pituitary receptors during preovulatory days due to low GnRH exposure	stable
			rapid downregulation of pituitary receptors during the ovulatory surge due to GnRH overexposure	
Healthy postmenopausal women	normal	secretion	low secretion due to estrogen deprivation	not applicable
		receptors	increased pituitary receptors due to low GnRH exposure leading to high FSH and LH secretion	
Anorexia nervosa	low	secretion	minimal secretion	
		receptors	normal or increased pituitary receptors	

It is important to note that shifts in GnRH secretion are expected only in women, they happen in lutheolysis and preovulatory and are compensated by changes in the availability of GnRH receptors within the following days. This might explain the unique susceptibility to downregulation of the GnRH pituitary receptors.

In males after the puberty onset, stable GnRH secretion lasts for decades (Table 1). Interactions of GnRH and gonadotropins with testosterone and inhibins are explained as simple control loops of negative feedback. Although due to presence of hypothalamic androgen receptors [2] direct androgen effects on male hypothalamus are expected, some estrogen is also locally produced from testosterone and this can act as an important regulator of GnRH secretion, similar as in women.

If we take gonadotropins more closely, the regulation of LH secretion seems simple. In men, it depends on testosterone action on the pituitary and on the previously mentioned combined testosterone/estrogen exposure that modulates hypothalamic GnRH secretion. In women it seems mainly regulated by progesterone exposure, although during the follicular phase it is regulated through GnRH actions with FSH.

FSH regulation is more complex. In men, beside the combined testosterone/estrogen modulation of hypothalamic GnRH secretion, FSH is regulated on the pituitary level by inhibin secreted from Sertoli cells [1,2]. In women, beside hypothalamic GnRH action, FSH depends on several hormones that act on the pituitary level. These are estrogen, inhibin A and inhibin B, activin etc. In describing actions of these regulators of FSH secretion, several issues seem important:

Estrogen binds to the pool of sex hormone binding globulin [1,2] and this binding alters estrogen availability to target tissues, particularly when this pool is empty, as happens during the follicular phase. This might explain the importance of inhibin B in preventing multiple ovulations [11]

inhibin A is secreted during the lutheal phase when it reduces FSH secretion [12] and thus limit the recruitment of primordial follicles for maturation in the following cycles. This might be related to the statements in Boron & Boulpaep [1] that *“…the monthly cycle of folliculogenesis actually begins from the primary-follicle stage 2 to 3 days before onset of the menses of the previous cycle. At this time, FSH levels begin to increase because of decreasing inhibin concentrations, thus inducing folliculogenesis, which is completed in the next cycle.”*.

activin is probably another fine-tuning modulator of the FSH secretion.

#### Comparison of ovulatory cycle and puberty

Regular shifting between the low and the high mode of GnRH secretion each two weeks in ovulating women resembles the puberty onset in several aspects (Table 1). Although some GnRH is secreted in childhood, prepubertal pituitaries in boys and girls seem to remain refractory until puberty [1,2] and this low GnRH secretion rate seems analogous to the follicular phase GnRH secretion.

Several mediators are involved in puberty initiation when GnRH secretion increases in both genders, particularly leptin and kisspeptin [13]. Since preovulatory changes in GnRH secretion are related to the increased exposure of hypothalamus to circulating estrogen, it seems plausible that hypothalamic estrogen exposure might be a decisive factor of entering puberty in both genders after priming with leptin, kisspeptin and other factors.

### Interplay between sex hormone receptors and ligands

#### Estrogen exposure increases expression of progesterone receptors

From breast cancer studies [14-19], it is well established that progesterone receptor (PgR) expression in cells with estrogen receptors (ER) depends on estrogen exposure during previous days. Similar effect of estrogen administration on pituitary PgR expression has also been reported [20].

Based on reports on various estrogen and progesterone target tissues (human uterus [21], endometrial hyperplasia [22,23], rat hypothalamic and limbic nuclei [24], anterior pituitary of rats [25,26], target organs in the female rat [27-29]), it is here proposed that without binding of various ER ligands (estradiol (E2) and probably some estrone (E1) locally converted from androgens), PgR expression remains low in pituitary and hypothalamus.

An indirect evidence for this proposition might lie in the known fact that medium and high dose progesterone-only contraceptive pills can prevent ovulation, while this cannot be achieved with low doses of progesterone alone [30]. This is also mentioned in Boron & Boulpaep [1]: *“…estrogens exert negative feedback at both low and high concentrations, whereas the progesterins are effective only at high concentrations"*. A possible explanation is that during the follicular phase a sufficient progesterone action is needed to prevent ovulation. Without estrogen action that increases expression of pituitary PgRs, available receptors are scarce and this lack of PgRs makes gonadotropin secreting cells less sensitive to progesterone exposure.

#### Progesterone exposure reduces both ER and PgR expression

It is mentioned in Boron & Boulpaep [1] that “ *…progesterone may act centrally by inhibiting gonadotropin secretion. Progestins are also antiestrogens. As a result, progestins acting locally may downregulate estrogen receptors and reduce the effectiveness of estradiol.*” Several reports show that progesterone exposure diminishes both ER and PgR expression in target tissues. Vereide et al. [29] have found in uterine mucosa that gestagen reduces glandular and stromal PgRs and ERs. Lundgren et al. [16] have reported that high dose oral gestagen diminishes PgRs, reduces ERs and androgen receptors in the breast cancer tissue.

Di Carlo et al. [26] showed that high doses of medroxyprogesterone acetate caused a very evident reduction in the weight of pituitary glands, suggesting that gestagens alter the hypothalamic-pituitary function.

### Proposed levels of regulation in the model

The model presumes that the GnRH shifting depends on simple interactions of ovulatory hormones and their hypothalamic receptors, although it might turn out to be much more complex in reality. It proposes that expression of hypothalamic and pituitary ERs and PgRs depends on binding of estrogen and progesterone to their receptors (Table 2). Estrogen exposure induces expression of new PgRs, while progesterone binding diminishes availability of both ERs and PgRs. This means that lutheolysis might be a consequence of progesterone binding that reduces expression of ERs. When hypothalamic sensitivity to estrogen is sufficiently reduced, hypothalamus switches back to the low GnRH secretion rate.

**Table 2:** The proposed interpretation of ligand interactions with their receptors

**Ligand exposure**	**Changes in receptor availability**		**Consequence of sustained ligand exposure**
	**ER availability**	**PgR availability**	
no ligands	increased	scarce	increased sensitivity to estrogen and reduced to progesterone due to lack of estrogen action
only ER ligands	normal or decreased	increased	estrogen action enhances progesterone sensitivity due to increased PgR expression
only progesterone	decreased	decreased	prolonged exposure to progesterone reduces sensitivity both to estrogen and progesterone due to reduced ER receptor synthesis that diminishes estrogen action and thus reduces PgR expression
ER ligands & progesterone			

Estrogen action on estrogen receptors (ER) is variable and acts as a prerequisite of progesterone receptor (PgR) expression. On the other hand, the progesterone actions are inherently limited, since sustained progesterone binding reduces both PgR and ER availability, leading to reduced sensitivity both to estrogen and progesterone.

**At the hypothalamic level,** the GnRH secretion rate depended on the detection of circulatory estrogens, so increased presence of hypothalamic ER due to low estrogen levels during the early follicular phase allows detection of rising estrogen secretion from growing follicles. As previously described, sufficient estrogen exposure shifts GnRH secretion to the high rate and this change is not the “positive feedback” phenomenon, since the high GnRH secretion rate is stable and lasts several days, much longer than the ovulatory surge.

During the lutheal phase, due to E and Pg exposure, hypothalamic ER and PgR are diminished until the estrogen exposure becomes undetectable. This change in hypothalamic sensitivity to circulating estrogens shifts the GnRH secretion rate to the low secretion that causes rapid lutheolysis.

**On the pituitary level,** gonadotropin secreting cells depend on GnRH action. Since the pituitary GnRHRs are increased during the follicular phase, increased GnRH secretion will maximally increase gonadotropin secretion until GnRHRs are spent and this partial downregulation of pituitary GnRHRs limits duration of the gonadotropin surge.

## Description of the proposed ovulatory cycle interpretation

This description is based on phase plane patterns from Graphs 2 and 3. Since precise blood values of other ovulatory cycle hormones are not available, the model is deliberately simplified to involve only these four hormones, namely FSH with estrogen and LH with progesterone. This model also lacks possible influences of estrogen, progesterone and activins on sensitivity of the pituitary gland to GnRH probably via changing the number of pituitary GnRH receptors. Nevertheless, all omitted plausible or possible interactions are not expected to contradict the here proposed simplistic model. In fact, it is expected that actions of in the model omitted hormones are not important for the proposed mechanisms that explain the gonadotropin surge and lutheolysis, based on these three key relations:

Hypothalamic GnRH secretion is increased under estrogen exposure during two weeks that start before the ovulatory surge and lasts till lutheolysis.

The pituitary GnRH receptors are so prone to downregulation through ligand binding that this must be important for their function.

In several estrogen target tissues, PgR expression depends on previous estrogen binding to functional ERs, while Pg binding to the expressed PgRs reduces both ER and PgR expression.

### The follicular phase

During the first four days (days of menstrual bleeding), the pool of estrogen binding proteins slowly releases estrogen accumulated from the previous cycle. In the next days, levels of inhibin, E and Pg are low, allowing FSH to rise. Early estrogen secretion cannot suppress the FSH secretion since most of the new estrogen is caught by the almost empty pool of sex hormone binding globulin (SHBG). Excess FSH secretion that might lead to multiple ovulations is prevented by inhibin.

On the day −7, new follicles start producing more estrogen and inhibin B. From the day −6 to −2, due to estrogen secretion from the dominant follicle, estrogen rapidly rises. Despite expected suppression, FSH is only slightly reduced, showing absence of the negative feedback of FSH with estrogen. A possible interpretation is that secreted estrogen binds to the unsaturated pool of plasma binding proteins. Inhibin B is probably important in preventing multiple ovulation due to inhibition of FSH secretion.

Between the day −3 and −1, estrogen action in the hypothalamus is strong enough to switch the GnRH secretion rhythm to a more intense level that forces rapid rises of FSH and LH secretions during one or two days, peaking on the day 0. The FSH peak is limited by pituitary exposure to estrogen and inhibin A & B through the negative feedback. The LH surge seems limited only by the GnRH action, since Pg level in the blood is low and the pituitary PgRs are still scarce. Expression of PgR was low due to low estrogen exposure during the previous week. The LH surge can be used as a measure of increased GnRH secretion since, median LH values reported by Stricker et al. [5] are 5.8 times increased (based on 41.19/7.02 from the reported data). This ratio is much lower in the FSH secretion due to pituitary sensitivity to estrogen and inhibins.

Intensified GnRH secretion rapidly downregulates the pituitary GnRH receptors and this reduction in pituitary GnRH sensitivity diminish both gonadotropin surges. Based on median values reported by Stricker et al. [5], the LH surge decline can be explained as a profound downregulation of GnRH receptors, leaving only some 36% of preovulatory pituitary GnRH receptors present after downregulation (based on 14.92/41.19 x100, from the reported data).

In short, ovulatory surges are here not explained by the “positive” feedback. Instead of that, the low GnRH secretion during the follicular phase allows the pituitary GnRHRs to accumulate. When the GnRH secretion is qualitatively increased, due to estrogen exposure, temporary gonadotropin surges happen. They last until the GnRHR downregulation returns the gonadotropin secretion under the negative feedback control. So, instead of mysterious “positive feedback”, here presented model proposes serial hormonal actions:

Early follicular phase:

low estrogen exposure increases pool of hypothalamic ERs

low GnRH secretion increases pool of pituitary GnRHRs

Late follicular phase

hypothalamus detects estrogen secretion and changes GnRH secretion to the high rate

Ovulatory surges

surges of gonadotropins last until pituitary GhRHRs are downregulated

the FSH surge is shaped by estrogen and inhibin negative feedback

the LH surge is maximal since pituitary is insensitive to low progesterone exposure due to low pituitary PgR expression

### Ovulation

The dominant follicle ruptures and subsequent local bleeding interrupts estrogen production. In the first postovulatory day (day +1), beside FSH and LH that fail due to downregulation of pituitary GnRH receptors, estrogen levels also fall due to interrupted ovarian secretion, but this fall is buffered by liberating some of SHBG bound estrogen.

### The lutheal phase

#### FSH & estrogen

For FSH secretion, a new balance between GnRH secretion, pituitary GnRH sensitivity on one and estrogen (and inhibin A) exposure on the other side is reached on the day +3. Between days +3 and +9, a negative feedback between FSH and estrogen is established, during which increased estrogen secretion reduces the FSH secretion.

#### LH & progesterone

Delayed estrogen action on pituitary and hypothalamic levels (started on days −7 to −2) leads to the delayed PgR expression and results in the lack of clear negative feedback between LH and progesterone. It is evident between days +1 and +4, when progesterone level strongly rise, despite slowly decreasing LH levels. On the day +5 to +9, the negative feedback of LH and progesterone steps in, LH drops due to progesterone action. Pg levels peak on day +7 and after that both Pg and LH decline.

### Lutheolysis

On the day +10, a small drop of FSH is followed by a huge drop of estrogen on the next day, showing that the hypothalamic switch to the low GnRH secretion rate has just happened. Lutheolysis happens on days +11 to +14. LH secretion hits the bottom on the day +11, and progesterone drops almost to the nonexistent levels at the cycle end. Simultaneously reduced estrogen (and probably inhibin A) levels allow some increase in FSH secretion, despite low GnRH secretion rates, suggesting initial recovery of pituitary GnRH receptors.

## Possible implications of the proposed interpretation on female and male reproductive physiology

The presented simplified ovulatory cycle model is possibly related to several topics in reproductive physiology.

### Possible actions of adrenal androgens in women

When considering possible roles of androgens in women, androgens on the pituitary level act twice: via androgen receptors, androgens regulate the LH secretion, but also and after being locally transformed to E1, they indirectly regulate the FSH secretion. So, increased extragonadal androgen exposure can block follicle maturation and prevent ovulation by suppressing FSH and LH surges, and thus can lead to polycystic ovaries.

Several entities seem linked to the role of adrenal steroids in women:

**Anorexia nervosa.** Low gonadotropin levels are reported in this disorder [30]. It is possible that diminished fat reserves do not secrete enough leptin and not convert enough adrenal androgens to estrogen. A combined estrogen and leptin deprivation reduces GnRH secretion, resembling a prepubertal situation [31] in which hypothalamus becomes refractory to all stimuli, if the body fat is very low.

**Menopause.** In menopausal women, adrenal androgens are converted to estrone in the adipose tissue. Postmenopausal estrogen action is to low to to suppress the FSH secretion, or to enhance the hypothalamic GnRH secretion. Since GnRH secretion remains low, pituitary GnRH receptors accumulate and thus increase the pituitary sensitivity to GnRH. Despite low GnRH secretion rate, due to increased pituitary sensitivity LH and FSH blood values are increased to the menopausal range. Beside that, low estrogen exposure of various target tissues lead to an increased ER and diminished PgR expression, making them more vulneable to estrogen related neoplasms, due to unopposed easrogen actions on them.

### Possible differences between the “female” and “male” hypothalamus

It is well recognized that the “male” hypothalamus is not able to generate ovulatory FSH and LH surges. Here presented interpretation proposes that the “female” hypothalamus is programed to act in two distinct modes of GnRH secretion. The low secretion period lasts from lutheolysis (day +10), till the late follicular phase (day-2), while the high rate period starts from the preovulatory phase (day −1) to lutheolysis (day +9). This two-mode mechanism allows increased pituitary sensitivity to GnRH on the day 0, responsible for the ovulatory surge. On the other hand, lutheolysis is caused by reduced hypothalamic sensitivity to estrogen that develops at the end of the lutheal phase, due to several days of progesterone exposure that diminishes both ER and PgR expression.

This interpretation also relies on the available pool of sex hormone binding globulin (SHBG). Presence of a large SHBG pool would postpone ovulation since estrogen secretion first saturates SHBG before exerting a decisive action on the hypothalamic level. This agrees with reports that SHBG synthesis in liver is augmented by estrogen and reduced by androgens, so in males a small and stable SHBG pool is expected, while in women the SHBG availability depends on estrogen action in the previous cycle.

### Anastrasole and gonadal disorders

Anastrosole, an aromatase inhibitor used in the treatment of ER positive breast cancer, is also used to control gynecomastia in boys [32] and in hypogonadic male adults [33]. In older men anastrasole can increase both testosterone and gonadotropin levels by reducing estrogen formation in adipose tissues. This can be explained if exposure to extragonadal estrogen was so intense that it gradually increased the GnRH secretion rate, until the GnRH pituitary receptors were so much downregulated that this resulted in low gonadotropin and testosterone levels.

Extremely obese male patients often have hypogonadism with low gonadotropin levels [34] and anastrasole can normalize gonadotropin secretion by inhibiting tissue aromatase. A plausible interpretation is that this disorder is caused by hypothalamic overexposure to extragonadal estrogen that alters GnRH secretion until downregulation of pituitary GnRH receptors diminishes gonadotropin secretion. This would result in a setting analogous to the castration by using a GnRH analogue.

## Evolutionary aspects of the proposed ovulatory cycle interpretation

Potential evolutionary survival advantages of interactions proposed in the described ovulatory cycle model are here briefly outlined. Since in many mammals daytime light exposure initiates estrous cycles in which ovulation is directly linked to the intercourse, conception and pregnancy are almost inevitable. The lack of seasonal reproduction in our *species* might suggest that we have evolved somewhere near the equator, where the lack of seasons could make the pineal gland control unimportant in our reproduction. Without seasonal estrous type reproduction, the probability of pregnancy declined sharply in our ancestors, leading to regular ovulatory cycling and frequent intercourses unrelated to ovulations. This evolutionary pressure for frequent ovulations forced our *species* to develop regular 28-day ovulatory cycles, probably as short as possible to allow pregnancy. The result is that reproduction of our *species* started to depend on the ability of our female ancestors to have regular ovulations and intercourses whenever it was energetically feasible.

In other words, it became unavoidable to our female ancestors to reproduce only during periods of normal food availability [35]. We can assume that in variable long periods of near starvation, surviving of our female ancestors required a transitory phase of hypogonadism, analogous to the endocrine setting seen in anorexia nervosa. This metabolic control over female reproduction is mediated through leptin action on the hypothalamus. During periods of low leptin exposure, no chances of pregnancy exist due to absence of ovulations and reduced libido. Energy and protein sparing in all estrogen target tissues, particularly in breasts and uterine mucosa enhance chances of survival during the harsh period.

With normal food availability, the whole system recovers. If no pregnancy has occurred within the optimal time frame of the lutheal phase, the lutheal progesterone secretion turns down both ER and PgR expression and leads to lutheolysis and further cycles.

A separate evolutionary issue is the presence of several regulators of FSH secretion in women. From the evolutionary perspective each mediator of FSH secretion during the ovulatory cycle has to have some survival advantage for our ancestors, otherwise it would probably be lost during evolution.

It seems plausible that mediators of FSH secretion function as optimizer of continuos cycling. For instance, to many primordial follicles are recruited in each ovulatory cycle, the ovarian reserves can be quickly depleted. If we are considering some 500.000 primordial follicles at the puberty age, a normal woman would spend some 1000 follicles in each cycle. So, the primordial follicle recruitment has to be limited to allow more ovulations. This is a potential role of inhibin A that tunes down the FSH secretion in the lutheal phase and thus spare some primordial follicles from activation. The other issue is that if more than one follicle matures, this might lead to dangerous multiple pregnancies that result in risky delivery of immature babies. Evolutionary response was the inhibin B action that tunes down the FSH secretion in the follicular phase and thus allows mainly single pregnancies.

An interesting consequence of this interpretation is that menopause might not be caused by the advantageous role of postmenopausal women in the care of their grandchildren, as proposed by G.C. Williams [36]. The main objection, mentioned by Williams in the cited paper [36] is that among our ancestors, very few women lived more than 40 years. Becoming postmenopausal must have been a rare event among our female ancestors. The expected few able grandmothers could not impose a substantial survival advantage that would result in the universal occurrence of menopause in all women after a certain age.

Due to these remarks, the menopause, as a strict gender specific feature of our *species*, needs to be explained within the frames of female reproductive physiology. Further, the increased importance of postmenopausal women in family life of our ancestors should be considered a consequence of physiology and not the cause.

Here proposed interpretation is that the ovarian pool of primordial follicles was optimized in our ancestors to allow sufficient chances of reproduction despite continuous ovarian activity. When sufficient reproductive probability was once achieved, there was no need for further enlargements of the primordial follicle pool in ovaries. After the average lifespan of our ancestors has become prolonged, the menopause has emerged because of normal depletion of ovarian reserves of primordial follicles.

## Competing interests

The author declares that he has no competing interests
